# Feasibility and acceptability of an online mindfulness-based group intervention for adults with tic disorders

**DOI:** 10.1186/s40814-021-00818-y

**Published:** 2021-03-24

**Authors:** Hannah E. Reese, W. Alan Brown, Berta J. Summers, Jin Shin, Grace Wheeler, Sabine Wilhelm

**Affiliations:** 1 Department of Psychology, Bowdoin College, 6900 College Station, Brunswick, ME 04011 USA; 2 Learning to Thrive, LLC, 300 2nd St., #7, Jersey City, NJ 07302 USA; 3 Center for OCD and Related Disorders, Massachusetts General Hospital, 185 Cambridge St., Suite 2000, Boston, MA 02114 USA; 4 Department of Psychology, University of North Carolina Wilmington, 601 South College Road, Wilmington, NC 28403-5612 USA

**Keywords:** Tourette syndrome, Tic disorder, Mindfulness, Treatment, Online, Group treatment

## Abstract

**Abstract:**

**Background:**

Preliminary research suggests that a mindfulness-based treatment approach may be beneficial for adults with tic disorders. In the present study, we report on the feasibility, acceptability, safety, and symptomatic effect of a novel online mindfulness-based group intervention for adults with Tourette syndrome or persistent tic disorder. Data from this study will directly inform the conduct of a funded randomized controlled trial comparing the efficacy of this intervention to another active psychological intervention.

**Methods:**

One cohort of adults with Tourette syndrome participated in an 8-week online mindfulness-based group intervention. Measures of feasibility, acceptability, and safety were administered throughout and at posttreatment. Self-reported measures of mindfulness and clinician-rated measures of tic severity and impairment were administered at baseline and posttreatment.

**Results:**

Data on refusal, dropout rate, attendance, participant satisfaction, and safety suggest that this is a feasible and acceptable intervention. However, participant adherence to home practice was lower than anticipated. Mindfulness, tic severity, and tic-related impairment only modestly improved from baseline to posttreatment. Qualitative analysis of participant feedback revealed aspects of the intervention that were most helpful and also areas for improvement.

**Conclusions:**

Data suggest that although this is a feasible and acceptable intervention, it should be modified to enhance participant adherence, more successfully engage the target mechanism, and optimize outcomes.

**Trial registration:**

Clinicaltrials.gov registration **#**NCT03525626. Registered on 24 April 2018

## Key messages regarding feasibility

- Preliminary research suggests that a face-to-face mindfulness-based intervention may be beneficial for adults with Tourette syndrome or persistent tic disorder. However, face-to-face psychosocial interventions for tics have been difficult to disseminate. Accordingly, we sought to further develop and test this mindfulness-based intervention in an online format that could be more readily disseminated to those seeking treatment. The present study was conducted to examine the feasibility, acceptability, safety, and symptomatic effect of the intervention prior to examining its efficacy relative to an active comparison condition within a randomized controlled trial. To our knowledge, this is the first test of an online mindfulness-based group intervention for adults with tics.
- Data on refusal, dropout rate, attendance, participant satisfaction, and safety suggest that this intervention is feasible and acceptable to adults with tics. However, participant adherence to home practice was lower than anticipated. Mindfulness, tic severity, and tic-related impairment only modestly improved from baseline to posttreatment. Qualitative analysis of participant feedback revealed aspects of the intervention that were most helpful and also areas for improvement.
- Participants were satisfied with many aspects of the intervention. However, data suggest that the intervention must be modified to boost participant adherence. We hypothesize that increasing participant adherence will produce more substantial changes in the proposed target mechanism (i.e., mindfulness), which will lead to more substantial symptomatic improvements.

Tourette syndrome (TS) and persistent tic disorder (PTD) are potentially disabling neurodevelopmental disorders characterized by sudden, rapid, recurrent motor and/or vocal tics [1]. The vast majority of individuals with TS or PTD also experience premonitory urges, which are uncomfortable sensations (e.g., a pressure, tension, or energy) that immediately precede the tics and are temporarily relieved by completion of the tics [2, 3]. Comorbid difficulties such as attention-deficit hyperactivity disorder (ADHD), obsessive-compulsive disorder (OCD) [4, 5], and disruptive behaviors (e.g., anger outbursts, aggression [6, 7]) are also common. Although prevalence estimates range, recent work suggests that TS and PTD combined affect approximately 1% of the general population [8, 9].

Although medications have long been the favored treatment approach for tics, psychosocial interventions for tic disorders have made great strides in recent years. For example, Comprehensive Behavioral Intervention for Tics (CBIT), an 8-session individual behavioral treatment, offers symptomatic relief comparable to that achieved with medication without burdensome side effects [10, 11]. Indeed, CBIT has recently been recognized as a first-line treatment option for children and adults with tics [12–14]. However, CBIT is still ineffective for a significant number of individuals [15, 16], and it has been challenging to disseminate [11, 17]. Accordingly, researchers have argued that we must continue to develop and test alternative psychosocial treatments, and especially those that can be readily disseminated [12]. In the present study, we report on the feasibility, acceptability, and symptomatic effect of a novel online mindfulness-based group intervention for adults with TS or PTD.

To date, mindfulness-based interventions have proven beneficial for individuals experiencing a wide range of psychiatric disorders [18]. We hypothesized that a mindfulness-based intervention could be beneficial to individuals with tics for several reasons.

First, mindfulness has been shown to improve attention regulation and body awareness (see [19, 20] for review). For individuals with tics, increased attention regulation and body awareness may facilitate awareness of urges to tic, tics, and factors that make the tics more or less likely to occur. This awareness is essential for accurate self-monitoring and informed self-regulation.

Second, and perhaps most importantly, mindfulness-based practices provide instruction and guidance regarding *being with* uncomfortable sensations, thoughts, and emotions, rather than immediately trying to change or escape them [21]. We hypothesize that this practice may help individuals cope with the discomfort of having urges to tic and tics. Current behavioral models of tics posit that tics are actions that the individual performs to alleviate the discomfort of the premonitory urge [22]. When the tic successfully rids the individual of the discomfort, it is negatively reinforced and so likely to be repeated. Through mindfulness practice, individuals may develop the capacity to gently observe and hold the discomfort associated with the urge thereby allowing it to subside without engaging in the tic. Over time, by allowing the urge to subside independently of the tic, the negative reinforcement cycle that is thought to maintain tics may weaken. This should, in turn, lead to a decrease in tics. This approach, although theoretically consistent with the model underlying CBIT, is a notable departure from CBIT in that it focuses on modifying how the individual responds to the urge rather than teaching the individual to stop the tic by engaging in an incompatible motor movement. Additionally, this capacity to tolerate the urge to tic may be particularly important in light of recent evidence suggesting that premonitory urges do not habituate with successful tic suppression [23, 24].

Third, mindfulness has been shown to reduce stress and improve emotion regulation [25, 26]. Many individuals report that stress and certain emotional states can provoke or worsen their tics [27]. It follows then, that reduced stress and improved emotion regulation may make tics less likely to occur or worsen. Similarly, there is robust evidence to suggest that mindfulness is helpful for anxiety and depression [28] and growing evidence to suggest that it may be helpful for obsessive-compulsive disorder [29] and attention deficit hyperactivity disorder [30]. Thus, it is reasonable to hypothesize that a mindfulness-based intervention may be helpful for comorbid conditions that frequently affect individuals with tics.

Finally, mindfulness-based practices encourage the adoption of a kind, accepting, and compassionate stance toward oneself [21]. This attitudinal shift toward kindness and acceptance may counter the negative self-perceptions often experienced by individuals with tics [31, 32] and potentially improve an individual’s well-being regardless of tic severity.

Two recent studies have provided empirical support to suggest that a mindfulness-based approach is worthy of further study. In one small open trial, adults with TS or chronic tic disorder who completed an 8-week, face-to-face, modified form of Mindfulness-based stress reduction (MBSR [33]) exhibited significant improvements in tic severity and tic-related impairment. Moreover, the percentage of participants deemed treatment responders exceeded that associated with CBIT in adults (58.8 and 38.1%, respectively [16]), and gains were maintained at 1-month follow-up. More recently, Gev et al. [34] conducted an experimental investigation comparing the effect of brief tic suppression instructions to brief urge acceptance instructions on urge frequency, intensity, and discomfort, as well as tic frequency among children with TS or PTD. The authors found that when individuals were instructed to notice and accept their urges, they experienced fewer urges, lower urge intensity, and less discomfort than when they were instructed to suppress their tics. Notably, there was not a significant difference between the tic suppression and urge acceptance conditions in degree of tic reduction. Thus, a mindful acceptance-based approach to tic urges produced short-term reductions in tic frequency that were comparable to suppression-focused approaches but with less urge-related discomfort.

While these studies show promise, face-to-face psychosocial interventions for tics have proven difficult to disseminate [35]. Patients report difficulty finding an accessible, trained provider in their geographic area, the time required for treatment, and the cost of treatment as barriers to treatment [35]. Thus, consistent with the National Institute of Mental Health recommendation to consider issues of dissemination in the earliest stages of treatment development [36], we sought to further study this approach by adapting it to be offered in an online, therapist-guided format that combines standardized, self-guided online instructional material, and live, therapist-guided discussion.

Online, therapist-guided treatments offer a number of advantages. First, they enable the delivery of treatments across great physical distance, improving the accessibility of trained providers. Second, they eliminate travel, reducing the time necessary for appointments. Third, they reduce cost by reducing the number of therapist hours necessary to deliver care. This is especially true in the case of the present intervention because it is being offered in a group format.

Evidence also suggests that online psychotherapeutic interventions are capable of producing benefits equal to face-to-face interventions for a range of conditions [37]. Evidence for the efficacy of online mindfulness-based interventions is also growing [38]. However, research on the efficacy of online treatments for individuals with tics is still in its infancy. Three pilot studies have examined the online delivery of behavior therapy to children with tics. In two of these studies, the online individual treatment was delivered in real-time via voice over internet protocol [39] and videoconference [40], respectively. In the third, more recent pilot study, the online individual treatment was delivered through asynchronous online self-help materials with written and phone support from a trained therapist [41]. However, to our knowledge, no studies have examined online treatments for adults with tics, who are typically less responsive to treatment. Moreover, no studies have examined the online delivery of a mindfulness-based intervention for children or adults with tics. And no studies have examined an online group-based intervention for children or adults with tics.

Thus, in the present study, our primary aim was to examine the feasibility, acceptability and safety of a novel, online mindfulness-based group intervention for adults with tics. We hypothesized that the intervention would be feasible and acceptable, as indexed by refusal, dropout, adherence, and participant satisfaction. We also hypothesized that the intervention would not be associated with adverse outcomes. Our secondary aim was to examine the effect of the intervention on self-reported mindfulness and clinician-rated measures of tic severity and tic-related impairment. We hypothesized that the intervention would result in increased self-reported mindfulness and decreased tic severity and tic-related impairment. Data from this study will directly inform the conduct of a funded randomized controlled trial (RCT) comparing the efficacy of this intervention to another active psychological intervention.

## Method

### Overview

Interested participants first completed a phone screen with the research assistant. Potentially eligible participants were then consented by the principal investigator and screened for eligibility by the Independent Evaluator (IE). Once enough eligible participants enrolled to form a group (consistent with Reese et al. [42], we aimed for a group size of 6 to capture a diversity of experiences, while still allowing for individualized attention and communication), participants completed a baseline assessment with the IE to establish tic severity and impairment in the week prior to beginning the intervention. The intervention lasted eight weeks and each week consisted of a 1.5-h self-guided online lesson, 1-h therapist-guided group videoconference, home practice, and questionnaires. Participants then completed a posttreatment assessment with the IE within 1 week of completing the intervention, and an exit interview with the intervention instructors within 1 month of completing the intervention. All study procedures were conducted by phone or videoconference via zoom.us, a secure online videoconferencing platform.

All study procedures were approved by the Bowdoin College Institutional Review Board (IRB), which served as the central IRB for this study, the Partners Human Research Committee, and carried out in accordance with the Code of Ethics of the World Medical Association (Declaration of Helsinki). All participants provided online consent prior to engaging in any study activities. The trial was registered at clinicaltrials.gov (Record #: NCT**03525626**).

### Participants

Participants were recruited via free online postings (Tourette.org, mghocd.org, clinicaltrials.gov) and emails to Tourette organizations, support groups, and providers (e.g., Tourette Association of America state and local chapters). Recruitment lasted approximately 1 month. To be eligible for the study, participants had to be 18 years of age or older, have a primary diagnosis of TS or PTD, be fluent in English, reside in the USA, and either not be taking any tic suppressant/psychotropic medication or be at a stable dose for 8 weeks prior to the baseline assessment and throughout the study. Participants were excluded from the study if they were receiving concurrent psychotherapy, had extensive prior experience with mindfulness and/or meditation, or had another psychiatric diagnosis or symptom (e.g., suicidality) that would necessitate additional clinical care.

### Assessment

Clinical assessments were conducted by an IE who was not otherwise associated with the study. The IE was a predoctoral psychology intern with experience conducting online assessments with clinical populations. The IE also received specialized training on the study measures from more experienced doctoral-level raters. Importantly, all of the assessments were conducted via videoconference which enabled the IE to directly observe participant tics. The IE then entered the interview data into Qualtrics, an online platform for electronic data capture that streamlines data collection and management and improves data quality. Self-report measures were sent directly to participants via Qualtrics.

### Measures^1^

#### Diagnostic evaluation

The Structured Clinical Interview for DSM-5 (SCID) is the gold standard in semi-structured diagnostic interviews to establish DSM-5 diagnoses [43]. The SCID was conducted at screening to assess for the presence of current or past DSM-5 disorders.

#### Adherence

Adherence to the intervention was indexed by two measures: 1) attendance at the weekly videoconferences and 2) amount of completed home practice. The amount of home practice completed was assessed via a self-report questionnaire on a weekly basis throughout the intervention. Participants were asked to report approximately what percentage of the formal and informal home practice they completed, on how many days they meditated, and approximately how many minutes, per day, they meditated.

#### Participant satisfaction

Participant satisfaction was assessed with the Client Satisfaction Questionnaire (CSQ [44]), an 8-item self-report scale (e.g., “How would you rate the quality of service you received? To what extent has our program met your needs?”). Each item is scored from 1 to 4 with higher scores indicating greater satisfaction. Total scores range from 8 to 32. The CSQ was administered at posttreatment to assess participant satisfaction with the intervention.

#### Qualitative feedback

Participants provided written qualitative feedback on a weekly basis throughout the program. Additionally, at the end of the study, the program instructors conducted individual qualitative interviews with each participant. The weekly feedback questionnaires and exit interviews focused on identifying aspects of the program that worked well, did not work well, and recommendations for improvement.

#### Safety

Consistent with recommendations to systematically assess the safety of mindfulness-based interventions [45, 46], adverse events (i.e., negative physical or psychological changes such as worsening of mood, anxiety, or tics, physical injury or pain) were assessed via self-report questionnaire on a weekly basis throughout the intervention and at posttreatment.

#### Mindfulness

Mindfulness was assessed with the Five Facet Mindfulness Questionnaire (FFMQ [47]), a widely-used 39-item self-report measure of five empirically derived facets of mindfulness: observing (e.g., “When I am walking, I deliberately notice the sensations of my body moving.”), describing (e.g., “I am good at finding words to describe my feelings.”), acting with awareness (e.g., “When I do things, my mind wanders off and I’m easily distracted.” reverse scored), non-judging of inner experience (e.g., “I criticize myself for having irrational or inappropriate emotions.” reverse scored), and non-reactivity to inner experience (e.g., “I perceive my feelings and emotions without having to react to them.”). Each item is scored on a 1 to 5 Likert scale and the scale yields an overall total score as well as the five subscale scores. Higher scores indicate greater mindfulness. The FFMQ was administered at baseline and posttreatment.

#### Tic severity and tic-related impairment

The Yale Global Tic Severity Scale (YGTSS [48]) is the gold standard clinician-rated instrument for tics. It has demonstrated excellent internal consistency, inter-rater reliability, and convergent and divergent validity [48]. The total tic score, which can range from 0 to 50, served as the primary symptomatic measure because it has demonstrated the greatest sensitivity to change in tic severity over brief periods of time [49]. The YGTSS impairment score, which can range from 0 (none) to 50 (severe), was also examined as a measure of tic-related impairment. The YGTSS was administered at baseline and posttreatment.

#### Improvement

The Clinical Global Impression-Improvement (CGI-I) Scale is a well-established seven-item clinician-rated measure of global improvement [50]. Consistent with other tic treatment outcome studies [15, 16], we defined treatment responders as those who received a rating of ‘much improved’ or ‘very much improved’ on the CGI-I. The CGI-I was administered at posttreatment only.

### Intervention

In developing the intervention, we adapted the modified MBSR curriculum developed by Reese et al. to make it more suitable for this population and mode of delivery while still ensuring that we retained the core features of MBSR [21]. Our adaptations were heavily informed by qualitative participant feedback from Reese et al. [42], and personal expertise of the member of the authorship team (W. A. Brown) who, in addition to being a mindfulness-based educator with extensive experience developing and teaching mindfulness-based programs in an online context, was also diagnosed with Tourette syndrome as a child.

Like MBSR, the intervention was 8 weeks long, involved approximately 2.5 h of instruction per week and focused on the cultivation of mindfulness through four primary meditative practices: the body scan, sitting meditation, walking meditation, and mindful movement. Also like MBSR, the practices were intended to help participants develop greater present-moment-focused attention, meet their experience with curiosity and compassion, recognize habitual patterns of the mind and body, and adopt a non-reactive, decentered stance toward their bodily sensations (and urges to tic, specifically), thoughts, and emotions.

In an effort to balance cost-effectiveness with prior research suggesting that guided online mindfulness-based interventions are more efficacious than unguided online mindfulness-based interventions [38], we chose to develop the intervention as a therapist-guided program, incorporating a self-guided online lesson and live therapist-led videoconferences. At the beginning of each week of the program, participants received an email with a link to the online lesson. They then logged in to the online lesson at their convenience. Each lesson consisted of a combination of audio and/or video recordings of guided mindfulness practices, self-guided inquiry (e.g., What did you notice in the practice that you just completed? How might this practice be relevant to working with your tics?), written or video-recorded didactics, and questionnaires. Each lesson required 1–1.5 h to complete, and participants could complete each lesson in one or several sittings. At the end of each lesson, participants were given a daily formal and informal home practice assignment. Formal practices consisted of guided meditation whereas informal practices encouraged the integration of mindfulness throughout one’s daily life (e.g., practicing mindful eating, mindfulness of pleasant events). Audio or video recordings and worksheets to support the home practice were provided to participants via a website. One week after the online lesson was sent out, participants met with each other and the two program instructors (HER and WAB) for a 60-min videoconference. During these videoconferences, the instructors led participants in discussion regarding their self-guided practice. In what follows, we provide a summary of the practices and intentions for each week of the program.

#### Week 1: an introduction to mindfulness

Participants were provided with a definition of mindfulness, and common misconceptions about mindfulness were addressed. They then completed ‘the raisin exercise’, a common introductory practice in which participants carefully attend to all five senses as they investigate and eat a raisin or other small object of food ([33], p. 27–28). Participants then read about the attitudinal foundations of mindfulness ([33], p. 33–40) and watched a video explaining the behavioral model of tics and the rationale for taking a mindful approach to tics. Finally, they completed a guided mindfulness of the breath meditation. Home practice included practicing mindful eating and a guided 10–20-min mindfulness of the breath practice once a day.

#### Week 2: mindfulness of the body

Participants began by practicing a guided body scan, a practice in which participants systematically, and nonjudgmentally attend to the physical sensations present in different areas of the body. They then practiced guided mindful movement consisting of gentle stretches and yoga while maintaining sustained and nonjudgmental attention to the body. Finally, they watched a video instructing them in the practice of mindfully attending to pleasant events (i.e., notice a positive experience and fully focus on it, noticing the senses as well as any associated thoughts and emotions). Home practice included practicing mindfulness of pleasant events and a guided 30-min body scan once a day.

#### Week 3: mindfulness of tics

Participants began by completing guided mindful movement consisting of stretches that could be completed while seated in a chair. They then began their first of three guided tic-specific seated meditations. These meditations were called “Riding the Wave” meditations and can be conceptualized as similar to urge surfing practices that have demonstrated efficacy within Mindfulness-based Relapse Prevention [51, 52]. The first of these practices focused on cultivating close, nonjudgmental attention to urges to tic, tics, and associated thoughts and emotions, without trying to change anything or behave any differently. Participants then watched an instructional video about walking meditation and completed the practice independently. Home practice included practicing mindfulness of unpleasant events and a guided 20-min Riding the Wave I seated meditation once a day.

#### Week 4: mindfulness of discomfort

Participants began the lesson with guided mindful movement (gentle yoga). They then watched a video explaining how tics can be connected to thoughts and emotions as well as bodily sensations. Next, they practiced the second guided Riding the Wave seated meditation which focused on noticing and exploring discomfort associated with the urges to tic and how one typically responds to that discomfort (e.g., making it go away, avoiding it, fighting it). Home practice included mindfully observing the thoughts, sensations, and emotions that precede and follow tics in daily life and practicing the guided 22-min Riding the Wave II seated meditation once a day.

#### Week 5: reacting vs responding

Participants again began the lesson with guided mindful movement (gentle yoga). They then watched a video discussing the difference between automatic habitual responding to stimuli and conscious, mindful responding. Next, they practiced the third guided Riding the Wave seated meditation. The third Riding the Wave meditation invited participants to practice being with or befriending discomfort (i.e., difficult premonitory sensations, related emotions, or thoughts) rather than automatically reacting to it. Home practice included looking for opportunities to mindfully respond rather than react in daily life, and practicing the guided 22-min Riding the Wave III seated meditation once a day.

#### Week 6: working mindfully with comorbid conditions

Participants began the lesson by practicing self-guided mindful movement. They then watched four videos discussing the relevance of mindfulness to working with symptoms of OCD, ADHD, anger, and irritability. They then practiced a guided Loving Kindness meditation focused on the cultivation of compassion toward others and oneself. Home practice included looking for opportunities to bring mindful attention to thoughts, emotions, or sensations associated with OCD, ADHD, anger and irritability, practicing the guided 14-minute Loving Kindness meditation twice, and practicing one of the previously practiced meditations (Riding the Wave, movement, body scan, etc.) on the remaining days of the week.

#### Week 7: making the practice your own

Participants again began the lesson by practicing self-guided mindful movement. They then practiced a mountain meditation ([53], p. 135–140), which uses the imagery of a mountain to facilitate non-identification with one’s thoughts, emotions, and sensations and the cultivation of a sense of strength and stability in the face of changing circumstances. Participants then began to consider how they would like to continue practicing mindfulness after the conclusion of the program by watching an instructional video and developing a written home practice plan for the week ahead.

#### Week 8: review and integration

Participants began the lesson by practicing an abbreviated form of the body scan. They then reviewed a written summary of the practices and concepts that they had learned in the prior weeks. They then practiced a final sitting meditation focused on mindfulness of the breath, body, sounds, thoughts, and emotions from a grounded, nonjudgmental, and kind perspective. Finally, participants developed a plan for continuing mindfulness practice after the conclusion of the program by writing about their intended actions, anticipated obstacles, and the supports necessary for success. To facilitate continued practice, participants were provided with a website that contained all prior mindfulness practices for streaming or download.

## Results

### Participant characteristics

Twelve individuals completed a brief phone interview to assess for eligibility. Five individuals were not eligible to participate, and one reported a scheduling conflict that precluded participation. Six individuals signed consent to participate in the study, completed the screening assessment, and were deemed eligible. One participant elected not to continue in the study for personal reasons. The remaining participants began treatment.

Of the five participants who were eligible for the study and began treatment, four were male, and one was female. The mean age was 39.6 years (*SD* = 13.1, *range*: 26–59). Four participants identified as white and one as Asian. One participant identified as Hispanic or Latino. Participants resided in five different states of the USA across three time zones.

All five participants met DSM-5 diagnostic criteria for a primary diagnosis of Tourette syndrome. Current comorbid diagnoses were as follows: obsessive-compulsive disorder (*n* = 4), attention deficit hyperactivity disorder (*n* = 3), generalized anxiety disorder (*n* = 1), social phobia (*n* = 1), agoraphobia (*n* = 1), specific phobia (*n* = 1), and hypochondriasis (*n* = 1). Concomitant medication status is presented in Table 1.

**Table 1:** Participant characteristics and treatment response

	FFMQ			YGTSS Total tic severity			YGTSS Impairment			
ID	Baseline	Endpoint	Change	Baseline	Posttreatment	Change	Baseline	Posttreatment	CGI improvement	Concomitant tic medication
1	129	130	1	39	39	0	30	30	Minimally improved	No
2	113	115	1	24	22	2	30	20	Much improved	Yes
3	112	135	23	34	29	5	40	30	Much improved	No
4	136	137	1	27	25	2	30	30	Minimally improved	Yes
5	119	--	--	32	32	0	30	30	No change	No

### Aim 1: feasibility, acceptability, and safety

#### Refusal and dropout

Only one individual chose not to participate in the study after completing the phone screen. That individual cited scheduling conflicts and asked to be re-contacted for future studies. One additional individual withdrew from the study after completing the screening assessment but before beginning treatment. That individual cited personal reasons. No participants dropped out after beginning treatment.

#### Adherence

Participants attended 87.5% of all scheduled videoconferences.

On average, participants reported completing between 41% and 60% of the assigned formal and informal home practice. To further assess the amount of formal practice completed, we compared the number of actual minutes of weekly meditation to the number of assigned minutes of weekly meditation. Across the first 6 weeks of the program (note that because participants devised their own home practice plans for weeks 7 and 8, we could not compare the actual to assigned home practice), participants were assigned an average of 108 min of meditation per week and completed an average of 39.7 min of meditation per week (*SD* = 16.3) which translates to a 36.8% completion rate.

#### Participant satisfaction

Scores on the CSQ can range from 8 to 32 with higher values indicating greater satisfaction. Scores on the CSQ ranged from 22 to 31, with a mean of 27 (SD = 3.87) indicating a high level of satisfaction with the intervention.

#### Safety

No serious adverse events were reported. One participant reported a knee injury. One participant reported a strained muscle. Two participants reported back pain. One participant reported food poisoning. None of these events were judged to be related to the intervention. Two participants reported transient worsening of tics during the study but did not demonstrate an overall worsening from baseline to posttreatment. Two participants also reported transient increases in anxiety and stress which they attributed to external factors.

#### Qualitative feedback

The qualitative questionnaires and interviews focused on identifying aspects of the program that worked well, aspects that did not work well, and suggestions for improvement. To analyze the qualitative data provided by participants, we adopted an inductive and comparative approach [54, 55]. We began by reviewing all participant responses to the self-report questions and exit interviews. We then identified statements directly relevant to our stated questions, coded them for meaning, identified repeating ideas, and arranged them into larger themes. Several themes emerged.

#### What was helpful

##### Increased awareness

Participants universally commented that they experienced increased awareness of their bodies, minds, and tics. This heightened awareness of “…what I am doing and how things impact me” led to greater self-understanding (“It [the practice] is a tool I can use to understand myself and my tics now and in the future”), an increased ability to notice tics as they are happening (e.g., “I am able to notice my tics sooner…”, “I think this could teach me to be aware of when (and maybe why) a tic is coming so I could adjust my mental and physical state”), an increased ability to notice the premonitory sensations associated with the tics (e.g., “I soon noticed that my tics ‘originate’ from my chest because I feel like a weight is on them.”), a recognition of factors that contribute to tic worsening (e.g., “Noticing that my tics get worse during times when I am tired and a bit hungry especially after 7 pm.”), and the ways in which one has typically responded to the urges and tics (“Ordinarily I suppress the urge to tic. I think usually that makes it more painful and sometimes more intense”).

##### Tools for becoming calm

Participants also frequently commented on the calming effect of the practices. For example, they stated, “an intense level of focus can help me recenter” and “I can get into a calm state pretty quickly”. “I continue to go to the breath awareness when I need a quick relaxing moment” and that the practices “calm me in stressful situations”. This calm state was described as being enjoyable and also typically associated with tic reduction. They liked having a practice that they could turn to when needing a respite from the tics (“This practice gives me the confidence that when things flare up and my tics get bad, I might have a practice to soothe and taper down the tics over a short period of time, to pivot and to just be again, with or without the discomfort”, “…the change is now when I feel things [tics and anxiety], I have a tool box of things I can turn to. This [the tics and anxiety] doesn’t have to be the rest of my day. I can get out of this and there are specific things I can do.”). One participant, who had previously received CBIT also stated that “meditation provided a relaxed and focused context for practicing [working with the tics] in contrast to CBIT in which I was supposed to practice in the midst of other activities.” Additionally, “…by being relaxed I can calmly explore the moment without always having the tics. It is harder when I am not calm. When I am doing the practice I am calm and I can better decide how to react to the urges to tic, compared to a normal moment in my day.” Thus, it seems that the calm experienced by the participants was directly related to tic reduction but also indirectly set the stage for working more mindfully with the urges to tic.

##### Acceptance and kindness

Participants also commented on the value of practicing acceptance and kindness. This was true as it pertained to working with discomfort (“This practice zooms in on the cycle and the discomfort associated with trying to not tic and I may get used to the sensation and thus not tic as much and just let the itch in my upper chest just be there and it may even shrink”), the tics themselves (“…accepting them [tics] with kindness and not judgment helps them dissipate quickly”) and the self (“This practice reminds me to be kind and forgive myself and others for parts of having Tourette’s that is unpleasant”). Participants noted a shift in their experience of the tics and their relationship to the tics (“I’m not sweating it [the tics] as much”) and a greater comfort with the self from this practice.

##### Group support and discussion

Participants universally commented on the value of the group support and discussion. Several participants had not previously had an opportunity to interact with other individuals with tics. They found the opportunity to share experiences, learn from others, and receive support to be integral to the treatment (“To do it without the connection-I don’t think it would really work as well.”) and meaningful (It’s “really important to know you’re not alone.”).

##### Specific practices

The Riding the Wave meditations and the mindful movement consistently emerged as being the most helpful practices for cultivating the changes described above. However, individual participants also cited other practices as being helpful and expressed an appreciation for having a range of practices to choose from.

#### What was not helpful

##### Technical issues

Several participants encouraged us to improve the production value and sound quality of our videos and the compatibility of the recordings with mobile devices.

##### Structural issues

Participants also frequently noted that they had difficulty completing the online lesson quickly enough to allow for home practice before the next week’s lesson was released. They also commented that sometimes it was not clear how the lesson and the home practice differed and when the two components should ideally be completed.

##### Content issues

A couple of participants noted that the lesson on comorbidities was too brief to be of benefit and could be eliminated to provide more time for working mindfully with the tics.

#### What improvements could be made

##### Psychoeducation

Several participants reported that they would like to have received more psychoeducation about tics, their causes, and how the practices connect to that scientific knowledge.

##### Home practice

Participants also expressed a desire for reminders or check-ins between the weekly videoconferences to help them stay on track with the home practice.

##### Assessment

Several participants expressed a desire for more open-ended and/or personalized questions in the outcome assessments. They often felt that the question anchors were limiting and that they wished they could elaborate or reflect more on their experiences. Examples of suggested improvements included an online journal, a qualitative question asking, “What is your relationship to your tics this week?” and the inclusion of a personalized weekly tic severity rating form.

### Aim 2: symptomatic outcomes

Because of the limited sample size, we elected to present participant-level scores in Table 1 rather than inferential statistics. Group means are provided below. With the exception of the FFMQ, all data were complete. One participant did not complete the FFMQ at posttreatment. That participant’s data are not included in the baseline to group mean comparisons, but they are included, when available, in Table 1. Two item-level responses to the FFMQ (representing 1.2% of all FFMQ item-level responses) were missing. These items were replaced with the response value closest to the mean subscale score for that item.

#### Mindfulness

At baseline, participants had a mean FFMQ total score of 122.5 (SD = 11.9, range: 112 to 136). At posttreatment, participants had a mean FFMQ total score of 129.3 (SD = 9.9, range: 115 to 137), representing a mean increase of 6.75 points (SD = 10.8). Examination of the subscale scores revealed that this increase in total mindfulness was largely accounted for by a mean increase of 6.0 points (SD = 3.37, range: 4 to 11) on the Nonjudge subscale. The remaining subscales demonstrated negligible change from baseline to posttreatment (Observe, *M* = 0, SD = 2.58, range: − 3 to 3; Describe, *M* = − .5, SD = 1.29, range: − 2 to 1; Act with awareness, *M* = .75, SD = 2.50, range: − 2 to 4; Nonreactivity, *M* = .5, SD = 2.38, range: − 1 to 4).

#### Tic severity and tic-related impairment

At baseline, participants had a mean YGTSS total tic severity score of 31.2 (SD = 5.9, range: 24–39) and a mean YGTSS impairment score of 32 (SD = 4.47, range: 30–40). At posttreatment, participants had a mean YGTSS total tic severity score of 29.4 (SD = 6.58, range: 22–39) and a mean YGTSS impairment score of 28 (SD = 4.47, range: 20–30). This represents a 1.8 point (SD = 2.04) mean reduction in tic severity and a 4 point (SD = 5.48) mean reduction in tic-related impairment.

#### Global improvement

Two participants were rated by the IE as ‘much improved’ on the CGI-I at posttreatment. The remaining participants were rated as ‘minimally improved’ or ‘no change’. Thus, 40% of participants were deemed treatment responders at posttreatment.

## Discussion

Overall, we found evidence to suggest that an online mindfulness-based intervention for tics is feasible, acceptable, and safe. We were able to recruit participants quickly and affordably and retain them in the study. Dropout was lower, and attendance was comparable to that seen in prior in-person randomized controlled trials of psychosocial treatments for adults with tics [16, 56]. This is especially noteworthy given the literature documenting high rates of dropout in internet-based psychological interventions [57]. Participants also reported being highly satisfied with the intervention. Indeed, the satisfaction scores were nearly identical to the scores obtained in the in-person pilot study of the intervention (scores ranged from 21 to 32; *M* = 27.8, *SD* = 3.7 [42];) suggesting that we have successfully adapted the intervention to an online format without sacrificing participant satisfaction. The intervention was also not associated with any adverse outcomes. However, we did notice a substantial gap between completed and assigned home practice. Because Reese et al. [42], did not report data on home practice completion, we cannot directly compare this online version of the intervention to the face-to-face version of this intervention. However, in a recent meta-analysis of home practice in MBSR and mindfulness-based cognitive therapy, Parsons et al. [58] found that participants completed approximately 64% of assigned practice, which is higher than the 36.8% completion rate that we observed. This would suggest that the current online intervention did not motivate engagement with the home practice to the degree observed in similar in-person interventions. We suspect that this is largely because of the timing and structure of the online lessons. In traditional in-person mindfulness-based intervention, new content is delivered within the weekly group meetings, and home practice is assigned for the intervening week. In the present intervention, new content was delivered in the self-guided online lesson, and home practice was assigned for the week between the release of the lesson and the live, online group meeting. Although the online lesson was available at the very beginning of the week, it was self-guided, and it sometimes took participants several days to complete it, leaving them with little time to complete the assigned practice before the live group videoconference. Going forward, we intend to alter the structure of the intervention so that new content is delivered in the live, online group meeting, and the home practice is assigned at the end of the meeting. We also intend to provide more psychoeducation to enhance the credibility of the intervention and develop personalized assessments to enhance participant motivation. Successfully improving adherence to the home practice will undoubtedly be a critical task for the subsequent RCT.

We also observed more modest symptomatic improvement relative to the in-person pilot study of the intervention. There are a couple of reasons why this may be. First, as noted above, the amount of home practice completed was much less than was assigned. It is possible that this reduced practice time could have contributed to the more muted symptomatic effects. Indeed, the meta-analysis by Parsons and colleagues [58] revealed a small, but significant (*r* = 0.26) correlation between home practice completion and clinical outcomes. Second, visual examination of the participant-level data in Table 1 reveal an interesting relationship between change in FFMQ scores and tic-related improvement. Mean values obscure the fact that only one participant (participant 3) showed a meaningful increase in mindfulness over the course of the intervention. That same participant is the only individual to exhibit a reduction in tic severity that is comparable to the reductions observed in the face-to-face pilot [42] and CBIT for adults [16]. Although we must interpret this very cautiously given the preliminary nature of this data, this relationship suggests that if we can modify the intervention to more successfully engage the hypothesized target (i.e., mindfulness), tic reductions may follow. Empirical examination of this hypothesis is, of course, necessary. It is also worth noting that the observed change in mindfulness was largely driven by the nonjudgment subscale. This is consistent with participants’ qualitative feedback emphasizing the value of increased acceptance and kindness. It also suggests that the intervention did not adequately target the hypothesized mechanisms of increased body awareness, attention regulation, and non-reactivity. This will also be a critical task for the subsequent RCT. And third, and relative to other psychosocial treatment studies of adults with tics, our sample was more severe at baseline. For example, in Wilhelm et al. [16], the mean baseline YGTSS severity scores were 24.0 for participants receiving CBIT and 21.8 for participants receiving Psychoeducation and Supportive Therapy. Additionally, participants with a YGTSS score above 30 were considered for exclusion. In contrast, our sample had a mean score above 30 with total scores ranging from 24 to 39. Indeed, the two participants with baseline scores above 30 are the two participants who did not demonstrate any changes in their YGTSS score. We intentionally did not restrict eligibility based on YGTSS scores to increase the generalizability of our findings. However, it is possible that tic severity moderates the efficacy of this treatment. It should be noted though, that prior work, examining moderators of psychosocial treatments for tic [59] would not support this hypothesis. This conjecture must be tested in future, larger studies.

It is also interesting that participants reported such high satisfaction with the intervention despite the relatively muted symptomatic changes. We suspect that the YGTSS did not fully capture the positive changes that may have occurred following the intervention. We saw some evidence of this in the discrepancy between outcome based on the YGTSS and the CGI-I. Although the reductions in the YGTSS ranged from 0 to 14.7%, two of five participants were rated by the IE as ‘much improved’ on the CGI-I (see Table 1 for details). This 40% response rate is comparable to that achieved with CBIT in adults [16]. However, prior research, examining the relationship between YGTSS improvement and CGI-I score [60] has suggested that an approximately 25% reduction in the YGTSS total score predicted a positive response (i.e., score of ‘very much’ improved or ‘much’ improved) on the CGI-I. This suggests that the perceived improvement that occurred in this study was not captured by the YGTSS. The improvement could be related to a sense of greater awareness, understanding, or control over the tics, the development of skills for coping with the urges and tics when they arise and/or a changing relationship to the tics such that they simply are not as bothersome or distressing although their number, frequency, intensity, complexity, and interference (i.e., the dimensions of tic severity assessed in the YGTSS) did not change. This is perhaps not that surprising since mindfulness-based interventions seek to change the individual’s response to their own experience rather than the experience itself. Individuals may be learning to understand and relate differently to their urges and tics. These changes would not be reflected in the YGTSS, but would certainly be meaningful outcomes from the practice. However, as mentioned earlier, the pilot study by Reese et al., [42] did demonstrate significant reductions on the YGTSS suggesting that the intervention may also be capable of producing symptomatic changes. Thus, future studies that ensure greater engagement with the intervention will be necessary to truly determine the potential symptomatic efficacy of this online intervention.

Taken together, these results suggest that this approach is feasible, acceptable, and safe for adults with tics. Further development and testing are clearly needed to enhance participant engagement and increase efficacy. Data from this study will substantially improve the next step in investigating this approach: a randomized controlled trial comparing it to another active psychological intervention. Should this approach prove beneficial, it could provide substantially greater access to an acceptable and effective treatment for the community of adults with tic disorders.

## Data Availability

The datasets generated and/or analyzed during the current study are not publicly available because consent to make the dataset publicly available was not obtained from the participants.

## Footnotes

1 A number of other measures were administered, but are not reported here. We recognized that this pilot study did not have enough participants to support analyses of multiple outcome measures. Nevertheless, we chose to administer these measures to get participant feedback on the process and perceived burden of completing them because they are slated to be administered within the RCT that will follow this pilot.

## References

[1] Association AP (2013). Diagnostic and statistical manual of mental disorders.

[2] Reese HE, Scahill L, Peterson AL, Crowe K, Woods DW, Piacentini J, Walkup JT, Wilhelm S (2014). The premonitory urge to tic: measurement, characteristics, and correlates in older adolescents and adults. Behav Ther.

[3] Scahill L, Leckman JF, Marek KL, Weiner WJ, Lang AE (1995). Sensory phenomena in Tourette's syndrome. Behavioral Neurology of Movement Disorders.

[4] Eapen V, Cavanna AE, Robertson MM (2016). Comorbidities, social impact, and quality of life in Tourette syndrome. Front Psychiatry.

[5] Freeman RD, Fast DK, Burd L, Kerbeshian J, Robertson MM, Sandor P (2000). An international perspective on Tourette syndrome: selected findings from 3,500 individuals in 22 countries. Dev Med Child Neurol.

[6] Robertson MM, Cavanna AE, Eapen V (2015). Gilles de la Tourette syndrome and disruptive behavior disorders: prevalence, associations, and explanation of the relationships. J Neuropsychiatry Clin Neurosci.

[7] Scahill L, Williams S, Schwab-Stone M, Applegate J, Leckman JF (2006). Disruptive behavior problems in a community sample of children with tic disorders. Adv Neurol.

[8] Robertson MM (2008). The prevalence and epidemiology of Gilles de la Tourette syndrome. Part 1: the epidemiological and prevalence studies. J Psychosom Res.

[9] Scahill L, Specht M, Page C (2014). The prevalence of tic disorders and clinical characteristics in children. J Obsessive Compuls Relat Disord.

[10] McGuire JF, Piacentini J, Brennan EA, Lewin AB, Murphy TK, Small BJ (2014). A meta-analysis of behavior therapy for Tourette syndrome. J Psychiatr Res.

[11] Scahill L, Woods DW, Himle MB, Peterson AL, Wilhelm S, Piacentini JC, McNaught K, Walkup JT, Mink JW (2013). Current controversies on the role of behavior therapy in Tourette syndrome. Mov Disord.

[12] Pringsheim T, Okun MS, Muller-Vahl K, Martino D, Jankovic J, Cavanna AE (2019). Practice guideline recommendations summary: treatment of tics in people with Tourette syndrome and chronic tic disorders. Neurology.

[13] Steeves T, McKinlay BD, Gorman D, Billinghurst L, Day L, Carroll A (2012). Canadian guidelines for the evidence-based treatment of tic disorders: behavioural therapy, deep brain stimulation, and transcranial magnetic stimulation. Can J Psychiatry.

[14] Verdellen C, van de Griendt J, Hartmann A, Murphy T, Group EG (2011). European clinical guidelines for Tourette syndrome and other tic disorders. Part III: behavioural and psychosocial interventions. Eur Child Adolesc Psychiatry.

[15] Piacentini J, Woods DW, Scahill L, Wilhelm S, Peterson AL, Chang S (2010). Behavior therapy for children with Tourette disorder: a randomized controlled trial. JAMA.

[16] Wilhelm S, Peterson AL, Piacentini J, Woods DW, Deckersbach T, Sukhodolsky DG, Chang S, Liu H, Dziura J, Walkup JT, Scahill L (2012). Randomized trial of behavior therapy for adults with Tourette syndrome. Arch Gen Psychiatry.

[17] Woods DW, Conelea CA, Walther MR (2007). Barriers to dissemination: exploring the criticisms of behavior therapy for tics. Clin Psychol Sci Pract.

[18] Goldberg SB, Tucker RP, Greene PA, Davidson RJ, Wampold BE, Kearney DJ, Simpson TL (2018). Mindfulness-based interventions for psychiatric disorders: a systematic review and meta-analysis. Clin Psychol Rev.

[19] Chiesa A, Calati R, Serretti A (2011). Does mindfulness training improve cognitive abilities? A systematic review of neuropsychological findings. Clin Psychol Rev.

[20] Holzel BK, Lazar SW, Gard T, Schuman-Olivier Z, Vago DR, Ott U (2011). How does mindfulness meditation work? Proposing mechanisms of action from a conceptual and neural perspective. Perspect Psychol Sci.

[21] Crane RS, Brewer J, Feldman C, Kabat-Zinn J, Santorelli S, Williams JM (2017). What defines mindfulness-based programs? The warp and the weft. Psychol Med.

[22] Woods DW, Piacentini J, Chang S, Deckersbach T, Ginsburg GS, Peterson AL (2008). Managing Tourette syndrome: a behavioral intervention for children and adults.

[23] Houghton DC, Capriotti MR, Scahill LD, Wilhelm S, Peterson AL, Walkup JT, Piacentini J, Woods DW (2017). Investigating habituation to premonitory urges in behavior therapy for tic disorders. Behav Ther.

[24] Specht MW, Woods DW, Nicotra CM, Kelly LM, Ricketts EJ, Conelea CA, Grados MA, Ostrander RS, Walkup JT (2013). Effects of tic suppression: ability to suppress, rebound, negative reinforcement, and habituation to the premonitory urge. Behav Res Ther.

[25] Wheeler MS, Arnkiff DB, Glass CR (2017). The neuroscience of mindfulness: how mindfulness alters the brain and facilitates emotion regulation. Mindfulness.

[26] Gu J, Strauss C, Bond R, Cavanagh K (2015). How do mindfulness-based cognitive therapy and mindfulness-based stress reduction improve mental health and wellbeing? A systematic review and meta-analysis of mediation studies. Clin Psychol Rev.

[27] Conelea CA, Woods DW (2008). The influence of contextual factors on tic expression in Tourette's syndrome: a review. J Psychosom Res.

[28] Khoury B, Lecomte T, Fortin G, Masse M, Therien P, Bouchard V, Chapleau MA, Paquin K, Hofmann SG (2013). Mindfulness-based therapy: a comprehensive meta-analysis. Clin Psychol Rev.

[29] Hale L, Strauss C, Taylor BL (2013). The effectiveness and acceptability of mindfulness-based therapy for obsessive compulsive disorder: a review of the literature. Mindfulness.

[30] Mitchell JT, Zylowska L, Kollins SH (2015). Mindfulness meditation training for attention-deficit/hyperactivity disorder in adulthood: current empirical support, treatment overview, and future directions. Cogn Behav Pract.

[31] Eddy CM, Rizzo R, Gulisano M, Agodi A, Barchitta M, Cali P (2011). Quality of life in young people with Tourette syndrome: a controlled study. J Neurol.

[32] Khalifa N, Dalan M, Rydell AM (2010). Tourette syndrome in the general child population: cognitive functioning and self- perception. Nord J Psychiatry..

[33] Kabat-Zinn J (1990). Full catastrophe living: using the wisdom of your body and mind to face stress, pain, and illness.

[34] Gev E, Pilowsky-Peleg T, Fennig S, Ben-Aroya-Milshtein N, Woods DW, Piacentini J (2016). Acceptance of premonitory urges and tics. J Obsessive Compulsive Relat Disord.

[35] Woods DW, Conelea CA, Himle MB (2010). Behavior therapy for Tourette's disorder: utilization in a community sample and an emerging area of practice for psychologists. Prof Psychol Res Pract.

[36] Onken LS, Carroll KM, Shoham V, Cuthbert BN, Riddle M (2014). Reenvisioning clinical science: unifying the discipline to improve the public health. Clin Psychol Sci.

[37] Barak A, Hen L, Boniel-Nissim M (2008). Shapira Na. A comprehensive review and a meta-analysis of the effectiveness of internet-based psychotherapeutic interventions. J Technol Hum Serv.

[38] Spijkerman MP, Pots WT, Bohlmeijer ET (2016). Effectiveness of online mindfulness-based interventions in improving mental health: a review and meta-analysis of randomised controlled trials. Clin Psychol Rev.

[39] Ricketts EJ, Goetz AR, Capriotti MR, Bauer CC, Brei NG, Himle MB, Espil FM, Snorrason Í, Ran D, Woods DW (2016). A randomized waitlist-controlled pilot trial of voice over Internet protocol-delivered behavior therapy for youth with chronic tic disorders. J Telemed Telecare.

[40] Himle MB, Freitag M, Walther M, Franklin SA, Ely L, Woods DW (2012). A randomized pilot trial comparing videoconference versus face-to-face delivery of behavior therapy for childhood tic disorders. Behav Res Ther.

[41] Andren P, Aspvall K, Fernandez de la Cruz L, Wiktor P, Romano S, Andersson E, et al. Therapist-guided and parent-guided internet-delivered behaviour therapy for paediatric Tourette’s disorder: a pilot randomised controlled trial with long-term follow-up. BMJ Open. 2019;9:1–9. 10.1136/bmjopen-2018-024685 PMC6398666 30772854

[42] Reese HE, Vallejo Z, Rasmussen J, Crowe K, Rosenfield E, Wilhelm S (2015). Mindfulness-based stress reduction for Tourette syndrome and chronic tic disorder: a pilot study. J Psychosom Res.

[43] First MB, Williams JBW, Karg RS, Spitzer RL (2015). Structured clinical interview for DSM-5-research version.

[44] Larsen DL, Attkisson CC, Hargreaves WA, Nguyen TD (1979). Assessment of client/patient satisfaction: development of a general scale. Eval Program Plann.

[45] Lustyk MK, Chawla N, Nolan RS, Marlatt GA (2009). Mindfulness meditation research: issues of participant screening, safety procedures, and researcher training. Adv Mind Body Med.

[46] Wong SY, Chan JY, Zhang D, Lee EK, Tsoi KK (2018). The safety of mindfulness-based interventions: a systematic review of randomized controlled trials. Mindfulness.

[47] Baer RA, Smith GT, Hopkins J, Krietemeyer J, Toney L (2006). Using self-report assessment methods to explore facets of mindfulness. Assessment.

[48] Leckman JF, Riddle MA, Hardin MT, Ort SI, Swartz KL, Stevenson J (1989). The Yale Global Tic Severity Scale: initial testing of a clinician-rated scale of tic severity. J Am Acad Child Adolesc Psychiatry.

[49] Lin H, Yeh CB, Peterson BS, Scahill L, Grantz H, Findley DB (2002). Assessment of symptom exacerbations in a longitudinal study of children with Tourette's syndrome or obsessive-compulsive disorder. J Am Acad Child Adolesc Psychiatry.

[50] Guy W (1976). ECDEU assessment manual for psychopharmacology.

[51] Bowen S, Witkiewitz K, Clifasefi SL, Grow J, Chawla N, Hsu SH, Carroll HA, Harrop E, Collins SE, Lustyk MK, Larimer ME (2014). Relative efficacy of mindfulness-based relapse prevention, standard relapse prevention, and treatment as usual for substance use disorders: a randomized clinical trial. JAMA Psychiatry.

[52] Witkiewitz K, Bowen S, Douglas H, Hsu SH (2013). Mindfulness-based relapse prevention for substance craving. Addict Behav.

[53] Kabat-Zinn J (1994). Wherever you go, there you are: mindfulness meditation in everyday life.

[54] Creswell JW (2013). Qualitative inquiry and research design: choosing among five approaches.

[55] Merriam SB (2009). Qualitative research: a guide to design and implementation.

[56] Deckersbach T, Rauch S, Buhlmann U, Wilhelm S (2006). Habit reversal versus supportive psychotherapy in Tourette's disorder: a randomized controlled trial and predictors of treatment response. Behav Res Ther.

[57] Melville KM, Casey LM, Kavanagh DJ (2010). Dropout from Internet-based treatment for psychological disorders. Br J Clin Psychol.

[58] Parsons CE, Crane C, Parsons LJ, Fjorback LO, Kuyken W (2017). Home practice in mindfulness-based cognitive therapy and mindfulness-based stress reduction: a systematic review and meta-analysis of participants' mindfulness practice and its association with outcomes. Behav Res Ther.

[59] Sukhodolsky DG, Woods DW, Piacentini J, Wilhelm S, Peterson AL, Katsovich L, Dziura J, Walkup JT, Scahill L (2017). Moderators and predictors of response to behavior therapy for tics in Tourette syndrome. Neurology.

[60] Jeon S, Walkup JT, Woods DW, Peterson A, Piacentini J, Wilhelm S, Katsovich L, McGuire JF, Dziura J, Scahill L (2013). Detecting a clinically meaningful change in tic severity in Tourette syndrome: a comparison of three methods. Contemp Clin Trials.

